# The Value of Coenzyme Q_10_ Determination in Mitochondrial Patients

**DOI:** 10.3390/jcm6040037

**Published:** 2017-03-24

**Authors:** Delia Yubero, George Allen, Rafael Artuch, Raquel Montero

**Affiliations:** 1 Clinical Biochemistry and Molecular Medicine Department, Institut de Recerca Sant Joan de Déu and CIBERER-ISCIII, Passeig Sant Joan de Déu, 2, 08950 Esplugues, Barcelona, Spain; dyubero@hsjdbcn.org (D.Y.); rmontero@hsjdbcn.org (R.M.); 2 Department of Blood Sciences, Royal Devon and Exeter NHS Foundation Trust, Exeter EX2 5DW, UK; george.allen@nhs.net

**Keywords:** coenzyme Q_10_ deficiency, mitochondrial diseases, treatment monitoring

## Abstract

Coenzyme Q_10_ (CoQ) is a lipid that is ubiquitously synthesized in tissues and has a key role in mitochondrial oxidative phosphorylation. Its biochemical determination provides insight into the CoQ status of tissues and may detect CoQ deficiency that can result from either an inherited primary deficiency of CoQ metabolism or may be secondary to different genetic and environmental conditions. Rapid identification of CoQ deficiency can also allow potentially beneficial treatment to be initiated as early as possible. CoQ may be measured in different specimens, including plasma, blood mononuclear cells, platelets, urine, muscle, and cultured skin fibroblasts. Blood and urinary CoQ also have good utility for CoQ treatment monitoring.

## 1. Introduction

Coenzyme Q_10_ (CoQ) is a lipid that acts in the mitochondrial respiratory chain (MRC) as the electron transporter from Enzymatic Complexes I and II to Complex III. Recognized biological functions of CoQ include an essential role in energy biosynthesis in the form of ATP, free radical detoxification, stabilization of mitochondrial enzymatic complexes, binding to the permeability transition pore, and the function of mitochondrial uncoupling proteins [1]. Almost all cells have the capacity for CoQ, and at least 13 genes have been shown to be required for endogenous production of CoQ. Furthermore, additional genes also influence CoQ availability such as those related with acetyl-CoA metabolism or those that can cause secondary reduction in CoQ biosynthesis or increase its degradation [2,3]. CoQ deficiency has been associated with different clinical phenotypes and genetic conditions [4] and environmental factors can also influence CoQ availability [5]. Regardless of the cause, the impairment of CoQ status can result in profound deficits to mitochondrial function. Treatment with CoQ supplementation can result in clinical improvement in CoQ deficiency, and early measurement of CoQ status is therefore of fundamental importance to allow the rapid initiation of treatment. Unfortunately, response to CoQ supplementation in trials with other mitochondrial disorders has been disappointing [6,7]. However, the lack of efficacy could potentially relate to delayed treatment; consequently, further studies are needed to ascertain whether early identification of CoQ deficiency in mitochondrial patients may help identify those in whom CoQ supplementation may yet prove beneficial [7].

## 2. Diagnostic Issues of CoQ Deficiency Syndromes

Currently, it is known that 8 of the 13 genes related to CoQ biosynthesis (*COQ* genes) can cause human disease [4], but these primary conditions are extremely rare. However, secondary CoQ deficiency is a common feature in a range of diseases. This susceptibility may be due to the intricate mechanisms and biological functions in which CoQ participates. Secondary deficiencies can occur in mitochondrial oxidative phosphorylation (OXPHOS) disorders [2,6,7] and in a broad spectrum of non-OXPHOS disorders [3]. Interestingly, the prevalence of muscle CoQ deficiency was demonstrated to be similar for both OXPHOS and non-OXPHOS disease patients [3]. Furthermore, it has been suggested that CoQ status may be an accurate predictor of deficient activity of MRC components [8,9], so routine CoQ measurement within the diagnostic workflow of OXPHOS disease seems advisable, especially for muscle biopsies.

Primary CoQ deficiency is considered a rare mitochondrial disorder associated with a heterogeneous clinical phenotype [4]. Nevertheless, clinical identification of potential cases is of paramount importance to initiate investigations that may provide early diagnosis and initiation of specific treatment, especially as some CoQ-deficient patients respond well to high oral doses of CoQ [10]. The clinical picture in primary CoQ deficiency can include ataxia with cerebellar involvement (the most common phenotype of CoQ deficiency syndromes), multiple organ failure in neonatal-onset forms, kidney disease, deafness, or muscular involvement [4], amongst others. The biochemical findings consist of a variable degree of CoQ deficiency in tissues (muscle/fibroblasts), which in turn may cause a reduction in the activity of the CoQ-dependent mitochondrial respiratory chain (Complexes I + III and Complexes II + III in muscle, Complexes glycerol-3-phosphate (G3P) + III and Complexes II + III in fibroblasts). However, it is not possible to biochemically distinguish between primary and secondary CoQ deficiencies nor to identify candidate genes for mutational analysis [11].

The initial stage of laboratory analysis is the biochemical identification of CoQ deficiency. Reduced activities of CoQ-dependent enzymes are indicative of CoQ deficiency, suggesting a decrease in electron transfer related to the quinone pool. This is supported by the restoration of Complex II + III activity after incubation with exogenous ubiquinone [12,13,14]. Nevertheless, direct quantitative measurement of CoQ levels is the most reliable test for diagnosis [15]. Essential to this is the choice of tissue for analysis. This may often be a balance between obtaining the most reliable sample for CoQ measurement and minimizing invasive procedures. However, some specimens such as plasma may not be suitable for the diagnosis of primary CoQ deficiency since misleading partial restoration of CoQ values from dietary sources of CoQ can occur in plasma. 

After establishing the biochemical diagnosis, the next step is to identify the specific genetic defect. Next-generation sequencing has largely replaced the need to serially sequence individual *COQ* genes and other genes associated with secondary deficiency and thus has profoundly changed the diagnostic process [11]. Nevertheless, biochemical measurements still play an important role in the diagnostic pathway by providing rapid and reliable demonstration of CoQ deficiency that allows early treatment initiation.

In this chapter, we will review the state of the art in CoQ measurement, utilizing different biological specimens for the investigation of mitochondrial disorders for both diagnosis and therapeutic follow-up. Additionally, we will highlight the advantages and pitfalls of CoQ determination in such specimens. 

## 3. CoQ Determination in Biological Samples. What Can We Expect?

CoQ is ubiquitously synthesized and found in almost all human cells, with higher CoQ concentrations found in organs with high-energy demand and metabolic rate. The measurement of both reduced and oxidized forms of CoQ allows for the determination of total CoQ, and this provides an optimal measure to detect CoQ deficiencies. CoQ levels in a range of specimen types from patients with mitochondrial diseases have been demonstrated to be lower than control values [6,7,16,17]. However, the particular CoQ distribution in distinct cellular fractions and the complexity of biological matrices makes the biological sample choice and preparation a critical step in the CoQ quantification process [18]. Additionally, since CoQ deficiency may be tissue-specific [19], invasive procedures are frequently needed in order to assess endogenous CoQ in the target organ, especially in muscle. Thus, it can be of value to analyze CoQ status in a full range of sample types, as a deficiency may remain undetected if the appropriate specimen is not chosen. Table 1 summarizes the different biological specimens and technical approaches for accurate CoQ determination. 

### 3.1. Blood Plasma 

Plasma CoQ is influenced by both dietary intake and hepatic biosynthesis [20]. Exogenous CoQ is absorbed through the gut by a complex process that can involve both active and passive mechanisms [21]. CoQ is then redistributed via the blood linked to cholesterol transporter lipoproteins [22] that act as the major carrier of CoQ in circulation [23]. The first step of patient CoQ estimation may be based on plasma measurement. However, CoQ status in plasma can be affected by both dietary supply and lipoprotein concentration. It is noteworthy that dietary sources of CoQ can significantly influence plasma CoQ concentrations, contributing up to 25% of the total amount [23]. For this reason, it has been suggested that plasma CoQ evaluation is not reliable for the diagnosis of primary CoQ deficiencies [24] as partial correction of CoQ levels may occur due to dietary consumption of CoQ or increases in cholesterol availability. Indeed, in most patients with primary CoQ deficiencies, plasma CoQ values are normal. Conversely, a reduction of plasma CoQ is not frequently observed in the general population, and although there is no demonstrated correlation of plasma and tissue CoQ values, decreased levels may reliably indicate secondary CoQ deficiencies associated with diseases such as phenylketonuria and lysosomal storage diseases [25,26,27,28,29,30]. Whether plasma CoQ can be used to indicate deficient tissue CoQ status in patients with mitochondrial disorders remains unknown at present.

While the usefulness of plasma CoQ analysis for the diagnosis of CoQ deficiency remains to be established, plasma CoQ determination has a critical role for CoQ treatment monitoring [31]. CoQ therapy is commonly used for the treatment of mitochondrial disorders and the follow-up of these patients should include regular plasma CoQ quantification. This allows for informed adjustment of the oral CoQ dose, the control of treatment compliance and confirmation of adequate CoQ intestinal absorption. The degree of distribution of this supplemented plasma CoQ from blood to affected tissues remains to be demonstrated and is still a matter of debate.

### 3.2. Blood Cells

Since analysis of plasma CoQ has limitations for diagnosis and avoidance of invasive first-step diagnostic procedures for the investigation of mitochondrial patients is desirable, a range of studies have tested the reliability of CoQ determination in different blood cell types. Remarkably, leukocyte CoQ levels correlate well with that of skeletal muscle and therefore represent a good alternative to evaluate endogenous CoQ [20]. Moreover, these cells can also display changes in cellular CoQ status upon CoQ supplementation [32]. This approach should be applicable for the identification of some patients with primary CoQ deficiencies. However, there is a lack of experience with these cells in most specialist clinical chemistry laboratories. Reference values have been reported [33], but no large-scale studies of mitochondrial diseases have been published. Unfortunately, the utility of this specimen currently remains constrained by technical limitations, including difficulties concerning sample collection and processing.

Similarly, platelet CoQ evaluation seems to be a good indicator of mitochondrial electron transport chain function [34,35]. Some studies have reported platelets as being a useful material for the determination of cellular CoQ content and of great utility for clinical monitoring CoQ treatment [36,37]. Although no reference values have been established, it is of note that platelet CoQ measurement may be advantageous compared to plasma during CoQ supplementation by providing a more representative measurement of cellular uptake and steady-state conditions [37]. However, even though detailed information about sample preparation and methods of detection are available, no studies of a large patient series have been published in relation to platelet CoQ evaluation for diagnosis or for follow-up in mitochondrial diseases.

Another possibility is to employ lymphoblastoid cell lines [11]. These cells combine the advantages and disadvantages of mononuclear cells and fibroblasts (see below) in that they do not require invasive procedures and allow for functional measurements. However, experience with these cells in clinical laboratories is very limited and the immortalization procedure may cause artifacts. In particular, immortalized cells tend to compensate the CoQ deficiency by overexpressing components of the CoQ biosynthetic machinery, so this may mask partial deficiencies.

### 3.3. Muscle 

Muscle biopsy material continues to be the best current option for investigations of CoQ status in mitochondrial disorders, with a biochemical diagnosis of CoQ deficiency indicated from the measurement of total CoQ content in muscle homogenates. The main advantage of this biological specimen is that other biochemical and histological studies may be conducted in parallel to evaluate the functional condition of the mitochondria, including measurement of mitochondrial respiratory chain enzyme activities, expression and assembly of mitochondrial complexes, and an assessment of the characteristic histopathological features of mitochondrial diseases [8]. Typically, a part of the muscle biopsy is processed in fresh conditions for histopathological and functional studies whilst the remaining sample is frozen immediately at −80 °C until analysis. The frozen samples are useful for CoQ determination and some other mitochondrial studies, with a minimum of 20–40 mg of muscle biopsy required for CoQ measurement [8]. 

Muscle CoQ deficiency is relatively common in patients with mitochondrial disorders [2,3,4,5,6,7,8]. Biochemically, it may be concomitantly present with decreased Complexes I + III and Complexes II + III activities, although other MRC complexes may be affected [8]. However, not all patients with muscle CoQ deficiency show MRC abnormalities, supporting the role of this molecule in other essential biological processes beyond energy production [2,17]. Some investigators have proposed CoQ redox status as a biomarker for oxidative stress [8,38]. However, this requires extensive and complicated investigations and is unnecessary to establish the diagnosis of CoQ deficiency.

In the last few years, a large list of mutated genes in patients who display secondary muscle CoQ deficiency has been reported [2,3,4,5,6,7]. All of these authors concluded that muscle CoQ deficiency is a frequent finding in mitochondrial disorders in general, with primary CoQ deficiency a much rarer condition. Clearly, some of these patients may benefit from CoQ supplementation aimed at restoring CoQ values and thus improving clinical outcomes [39]. Recently, it has been demonstrated that muscle CoQ values are a good predictor of MRC enzyme function with a utility at least equal to citrate synthase activity [9]. This provides added value for muscle CoQ analysis in the investigation of mitochondrial disorders. 

### 3.4. Fibroblasts

Skin fibroblasts are also a good specimen for demonstrating CoQ deficiency [40]. CoQ content and MRC enzyme activities can be measured in fibroblasts alongside different in vitro studies for assessing CoQ synthesis and other metabolic pathways [41,42]. Fibroblast CoQ deficiency may be accompanied by decreased activities of Complexes G3P + III and Complexes II + III that may or may not be associated with deficiency of other MRC complexes [17].

Fibroblasts are usually obtained from minimally invasive skin punch biopsies and after culturing in standard medium (Dulbecco’s Modified Eagle Medium (DMEM) containing 10% Fetal Bovine Serum (FBS) and 1% penicillin-streptomycin) [40], these cells are amenable to biobank storage, making them a very valuable material for future investigations. To analyze CoQ content, cultured skin fibroblasts are homogenized and lipids are extracted before CoQ quantification [43]. In addition, in vitro investigation of CoQ biosynthetic capacity can be performed in cultured fibroblasts. These assays are used to analyze the incorporation of radiolabeled substrates into CoQ, such as [3H]-mevalonate and [14C]-4-hydroxybenzoate, or that of stable isotopes with the measurement of synthesized CoQ by high pressure liquid chromatography (HPLC) with radiometric or tandem mass spectometry (MS–MS) detection [41,44,45]. These studies have been demonstrated to be very useful in discriminating between a primary CoQ deficiency, where the CoQ biosynthesis downstream of the provided substrates is impaired, and secondary CoQ deficiencies, where other mechanisms leading to CoQ deficiency are expected [41]. However, some limitations inherent to fibroblasts have been reported. For example, patients with muscle CoQ deficiency may show normal CoQ values in fibroblasts [46], and secondary fibroblast CoQ deficiency has been described in patients with other non-mitochondrial diseases [41].

Other investigations in cultured skin fibroblast have provided insights about key pathophysiological aspects implicated in CoQ deficiency, such as impairment of ATP synthesis or increased free radical damage [47,48]. Other pathophysiological mechanisms proposed include the implication of CoQ in pyrimidine biosynthesis (demonstrated in *COQ2*-mutant fibroblasts) [49] and the potential induction of mitophagy in response to CoQ deficiency [50]. Cotan et al. [51] reported that MELAS fibroblasts show a significant reduction of the mitochondrial membrane potential associated with secondary CoQ deficiency, which triggers mitochondrial degradation by mitophagy. Fragaki et al. [52] also observed a secondary mitochondrial dysfunction in ganglioside GM3 synthase (EC:2.4.99.9) deficient patients (including decrease in Complexes I + III and Complexes II + III in the liver among other MRC abnormalities in fibroblasts), leading to the impairment of normal mitochondrial electron flow and proton pumping, including a drop in mitochondrial membrane potential and an increase in apoptosis.

### 3.5. Urine 

Mitochondrial diseases can be associated with renal involvement. Interestingly, primary CoQ deficiency patients can present with a nephrotic syndrome either in isolation or in combination with other clinical signs [53,54]. This is perhaps not surprising given that renal tubules and glomerular podocytes are rich in mitochondria, allowing them to satisfy a high metabolic demand. Moreover, dramatic clinical improvement of patients with renal disease and CoQ deficiency has been observed following CoQ supplementation [10]. Urinary tract CoQ analysis could be an appropriate approach to assessing kidney CoQ status and may help fulfill the critical need for less invasive procedures to determine tissue CoQ status. Recently, a new methodology for the measurement of CoQ in urine has been standardized, including the establishment of reference values for a pediatric control population [55]. This new evaluation of urinary tract CoQ is a noninvasive procedure that might be useful for estimating CoQ kidney status for diagnosis and especially for CoQ treatment monitoring.

### 3.6. Other Biological Samples

CoQ is present in all tissues with its abundance in individual tissues associated with energy requirements or metabolic activity. Consequently, CoQ content displays a great variability between different organs and even between cells within the same organ [33]. Several authors have reported reduced CoQ levels in other tissues such as liver or kidney in patients with mitochondrial diseases [6,56,57]. However, these procedures require invasive biopsy procedures that can only be justified if other options for investigations of CoQ status have been exhausted. Experimental measurement of CoQ has also been reported in cardiac tissue obtained from patients undergoing heart transplantation [58]. This study found moderate decreases in CoQ in patients with heart failure in comparison to those without heart failure.

CoQ deficiency can present with profound neurological features, so measurement of CoQ in cerebrospinal fluid (CSF) has the potential to provide important clinical insight as an indicator of brain CoQ status. The concentration of CSF CoQ is in the low nanomolar range and therefore requires specialist analysis by tandem mass spectrometry [59]. Reference ranges have been established for CoQ in CSF, and a suggested application for this technique is in the identification of cerebral CoQ deficiency, although pathological samples were not reported [59].

Very recently, a new approach for CoQ determination has been described using non-invasive mouth swab collection of buccal mucosa cells for CoQ measurement by micro-HPLC. This technique may prove to be valuable for monitoring pediatric patients [60].

## 4. CoQ Quantification: Technical Aspects

**Figure 1:**
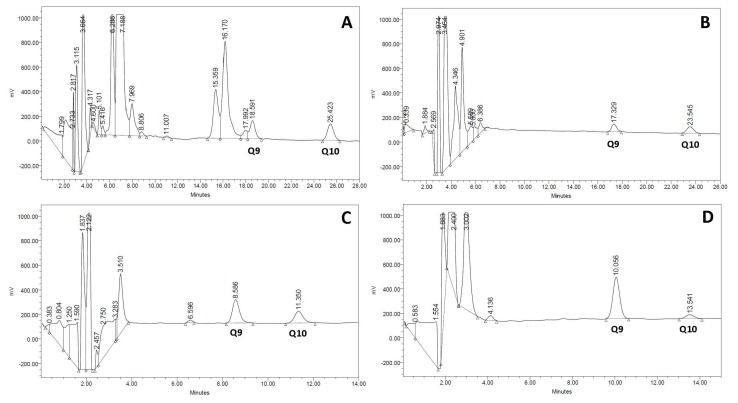
Normal Coenzime Q_10_ (CoQ) chromatograms of different biological specimens. (**A**) serum; (**B**) urine; (**C**) muscle; (**D**) cultured skin fibroblasts. In each specimen, type Q_9_ and Q_10_ have a different retention time that is related to differences in sample matrices and the high-pressure liquid chromatography (HPLC) column length required for separation.

The gold standard procedure for biochemical diagnosis of CoQ deficiency is the analysis of CoQ concentration by HPLC with ultraviolet or electrochemical detection [11]. Recently, new procedures for CoQ determination have been developed based on liquid chromatography-tandem mass spectrometry [41], allowing not only CoQ quantification but also an estimation of the CoQ biosynthetic rate in fibroblast cell cultures incubated with adequate CoQ precursors. Methodological approaches for CoQ measurement are reviewed in another chapter of this issue. Typical CoQ chromatograms from serum, urine, muscle, and cultured skin fibroblasts are depicted in Figure 1. 

## 5. Conclusions

CoQ is a molecule involved in multiple essential biological functions mainly within the mitochondria. The intricate metabolic pathways related to CoQ biosynthesis and metabolism underlie a vulnerability to frequent reductions in the concentration of this molecule as a consequence of different disease states, but are especially relevant in mitochondrial disorders. Because of this, the measurement of CoQ status in different biological specimens can be considered an essential part of the diagnostic and research workflows for patients with mitochondrial disorders and for CoQ treatment monitoring. This is particularly crucial as CoQ deficiency can be a treatable condition in some cases, so early recognition of the CoQ-deficient status is important to allow for the commencement of CoQ therapy as soon as possible.

## Figures and Tables

**Table 1:** Advantages and limitations for the CoQ analysis in different biological specimens.

Tissue	Advantages	Limitations
Plasma	Minimally invasive Identification of secondary CoQ deficiencies CoQ treatment monitoring	Low diagnostic yield for CoQ deficiency in mitochondrial disorders CoQ values modified by external sources
Leukocytes Platelets	Minimally invasive Correlation with CoQ tissue levels CoQ treatment monitoring	Fresh preparation Time-consuming Few reported experiences in mitochondrial disorders.
Muscle	Good diagnostic yield for CoQ deficiency Other mitochondrial studies can be performed	Invasive No treatment monitoring
Fibroblasts	Good diagnostic yield for some CoQ deficiencies Functional studies can be performed (CoQ biosynthesis) Unlimited biological material for further studies	False negative results in some cases
Urine	Non-invasive Easily detectable CoQ values Treatment monitoring purposes	Correlation with kidney CoQ status remains to be established

Note: Coenzyme Q_10_ (CoQ).
